# Under Development JAK Inhibitors for Dermatologic Diseases

**DOI:** 10.31138/mjr.31.1.137

**Published:** 2020-06-11

**Authors:** Nikolaos Sideris, Efstratios Vakirlis, Aikaterini Tsentemeidou, Alexandra Kourouklidou, Demetrios Ioannides, Elena Sotiriou

**Affiliations:** 1^st^ Dermatology Department, School of Medicine, Aristotle University, Thessaloniki, Greece

**Keywords:** Janus kinase inhibitors, atopic dermatitis, psoriasis, vitiligo, alopecia areata

## Abstract

Molecular targeting therapies represent a new exciting era in dermatology. A promising novel drug class, subject of intense research, is Janus kinase (JAK) inhibitors. Multiple cytokine receptors signal through the Janus kinase and signal transducer and activator of transcription (STAT) pathway. The pathway plays a central role in innate and adaptive immunity, and haematopoiesis. The understanding of the contribution of JAKs to the immunologic processes of inflammatory diseases led to the development of JAK inhibitors, initially for rheumatologic and hematologic diseases. Soon, their efficacy in some dermatologic conditions was also demonstrated, and today their role as therapeutic agents is thoroughly researched, mainly in atopic dermatitis, psoriasis, vitiligo, and alopecia areata. JAK inhibitors can be administered orally or used topically. As they are relatively new treatment modalities in dermatology, many questions concerning their efficacy and safety remain unanswered. Data from ongoing trials are eagerly awaited. Here, we summarize under development JAK inhibitors for dermatologic diseases.

## INTRODUCTION

Janus kinases (JAKs) is a family of intracellular tyrosine kinases consisting of four members (JAK1, 2, 3, and tyrosine kinase 2 [TYK2]). They are named after the two-faced Roman god because they are comprised of two phosphate-transferring domains with opposite roles. One domain exhibits, and the other inhibits the kinase activity.

JAKs, Signal Transducer and Activator of Transcription (STAT) proteins consisting of seven members, STAT-1,-2,-3,-4,-5a,-5b and -6, and trans-membrane receptors are the three main parts of the JAK-STAT pathway, which transmits extracellular information to gene promoters inside the nucleus.^1^

A vast array of hormones, interferons, colony-stimulating factors and interleukins exert their actions through the JAK-STAT pathway.^2^ Those factors, after binding to their corresponding receptors, activate JAKs, which consequently phosphorylate the receptors, allowing STATs to bind to them and become phosphorylated. Phosphorylated STATs migrate to nucleus where they affect gene expression.^3^

The importance of JAKs in human physiology, especially in immunity and haematopoiesis, was revealed quickly after their discovery and they became a field of intense research. The unveiling of their role in inflammatory^4–6^ and myeloproliferative^7,8^ diseases, identified JAKs as possible therapeutic targets. This led to the development of JAK inhibitors, a new and exciting era in pharmacologic development. Today, five JAK inhibitors are approved in USA and/or Europe, one only in Japan (peficitinib) for the treatment of rheumatoid arthritis (RA) and one (oclacitinib) for canine atopic dermatitis (AD) (*Table 1*). Many more are under development for various rheumatologic, dermatologic, neoplastic and other diseases.

**Table 1.** Approved JAK inhibitors.

DRUG	INDICATION	NOTE
*First generation*		
tofacitinib	RA, PsA, UC	
ruxolitinib	myelofibrosis, polycythemia vera	
baricitinib	RA	
oclacitinib	AD	only for dogs >12 months of age
*Second generation*		
fedratinib	myelofibrosis	
upadacitinib	RA	
peficitinib	RA	only in Japan

RA: rheumatoid arthritis, PsA: psoriatic arthritis, UC: ulcerative colitis, AD: atopic dermatitis.

Here, we summarize under development JAK inhibitors for dermatologic autoimmune/inflammatory conditions.

## ATOPIC DERMATITIS

Atopic dermatitis (AD) is a common inflammatory skin disease affecting as much as 25% of children and 10% of adults.^9^ Prevalence depends mainly on genetic and socio-economic factors, with developed countries being more affected. As the child ages, the disease improves or totally resolves in more than 50% of the patients over 6 years old, but in some cases, persists or even starts in adulthood.

The main characteristics of the disease are pruritus, eczematous lesions usually in age-specific body parts, dry skin and chronic course with relapses and remissions. AD imposes a substantial psychosocial burden on patients and their relatives. Pruritus and the accompanying sleep disturbance are not only distressing but also increase the risk for psychiatric conditions like ADHD, depression, suicidal ideation, autism, and others.^10^

Asthma, allergic rhinitis and food allergy are known associations of AD. Multiple other complications and comorbidities have been reported including, but not limited to, growth delay, bacterial and viral infections, ocular abnormalities, aortic stiffness, other allergic, metabolic and autoimmune conditions (Crohn’s disease, alopecia areata, vitiligo, etc.).^11,12^

Many key processes of AD pathogenesis, such as increased Th2 response, eosinophil activation, suppression of regulatory T-cells and structural factors of the skin, are due to the activation of the JAK-STAT pathway by numerous cytokines.^13^

### Ruxolitinib

Ruxolitinib is a JAK1/JAK2 inhibitor that, apart from AD, has been trialled for many other dermatologic (psoriasis, vitiligo, alopecia areata) and non-dermatologic diseases. One clinical trial (NCT03011892) comparing mean percentage change from baseline in Eczema Area and Severity Index (EASI) score at Week 4 in subjects treated with 1.5% ruxolitinib BID compared with subjects treated with vehicle cream BID has been completed in March 2018 but the results are not available yet.

Another three trials are currently active. NCT03257644 is a pharmacokinetic study in paediatric patients. NCT03745638 and NCT03745651 are phase 3, double-blind, randomized trials to access the efficacy and safety of ruxolitinib cream in adolescents and adults with AD. A total of 1200 patients are estimated to be enrolled in the last two studies.

### Delgocitinib

Delgocitinib is a topical pan-JAK inhibitor. Encouraging results firstly published by Nakagawa et al.^14^ who conducted a placebo-controlled dose-ranging study on 327 patients.

One more, phase 2b, double-blind, randomized, dose ranging trial to evaluate the efficacy and safety of delgocitinib cream in adults with AD started recently (NCT03725722), and another (NCT03826901) is expected to start soon to evaluate the pharmacokinetics of the substance.

### Tofacitinib

Only one phase 2 trial is published on topical administration of tofacitinib, a JAK1/JAK3 inhibitor, for AD,^15^ with very promising results. In 69 adults with mild-to-moderate AD, randomized 1:1 to 2% tofacitinib or vehicle ointment twice daily, change from baseline in EASI was 81.7 for tofacitinib vs. 29.9 for vehicle. All relevant scores (EASI, PGA and BSA) were improved by the first week of treatment and pruritus by day 2.

### Baricitinib

Baricitinib, as a JAK1/JAK2 inhibitor, modulates cytokines associated with both acute (JAK1: IL-4, -6, -10, -13, -31, IFN-γ / JAK2: IL-5, -6, -23, -31, IFN-γ, GMCSF) and chronic (JAK2: IL-23) AD lesions.^16^

Results of a phase 2 clinical trial have been published.^15^ According to those, 61% of the patients under baricitinib 4-mg achieved EASI-50 compared to 37% under placebo. Baricitinib also improved pruritus and sleep loss. A phase 3 trial (NCT03334422) with 750 participants has been completed recently with yet unavailable results and another six phase 3 trials are currently active.

Most frequently reported adverse events (AEs) of baricitinib are infections, low neutrophils, and increases in creatinine, transaminases, high- and low-density lipoproteins and creatine phosphokinase levels. Laboratory abnormalities are usually clinically insignificant and rarely lead to discontinuation of treatment.^17,18^

### Upadacitinib

Upadacitinib, a selective JAK1 inhibitor, is one of the most promising new drugs. In total, 35 trials are active or have been completed recently, for various diseases, as Crohn’s disease, RA, ankylosing spondylitis, giant-cell arteritis and others.

Nine of those trials concern AD. Only one has been completed. In this, patients were randomized to four arms (upadacitinib 7.5 or 15 or 30 mg or placebo) for 16 weeks. Change from baseline EASI was 39.4, 61.7, 74.4 and 23 respectively. Patients in the 30mg arm achieved the best results, EASI 90 in 50% compared to 2.4% in the placebo arm. Drug’s effect was fast, decreasing skin lesions by week two of treatment and pruritus by week one.^19^

Most frequent AEs were upper respiratory system infections. There was not a difference in severe AEs between the upadacitinib and placebo groups.

### Other JAK Inhibitors

Other JAK inhibitors in earlier stages of research in AD are abrocitinib (PF-04965842), a JAK1 inhibitor, and gusacitinib (ASN002) and cerdulatinib (RVT-502), inhibitors of both JAK and spleen tyrosine kinase (SYK) pathways. Concerning the possible parallel effect of JAK inhibitors on the multiple comorbidities of AD, not many data are available and no safe assumptions can be made at this time point. Nevertheless, a positive action not only on AD but also on some of its comorbidities would not be surprising.

For example, allergic diseases, which are often associated with AD, are primarily characterized by increased levels of IL-4, IL-5, IL-13, IL-31, and thymic stromal lymphopoietin (TSLP). All those cytokines induce the JAK/STAT pathway after binding to their respective receptors.^20^

There are also reports of positive impact of JAK inhibition on inflammatory-driven comorbidities of myelofibrosis and related neoplasms.^21^

JAK inhibitors may provide some benefit to patients with allergic diseases by specifically targeting inflammatory pathways important for disease pathogenesis, but current clinical trials are focused on AD, rather than on its allergic comorbidities or allergic diseases in general.

## PSORIASIS

Psoriasis is another common inflammatory pruritic skin disease, characterized by abnormal epidermal growth, usually presenting as red, scaly patches, papules or plaques (psoriasis vulgaris) with a prevalence of approximately 2–3%. Other phenotypes of the disease include guttate, inverse, pustular, generalized pustular, palmoplantar and erythrodermic psoriasis.^22^ 10 to 30% of the patients, depending on the study population, have concomitant psoriatic arthritis (PsA).

Psoriasis causes severe impact on quality of life, stigmatisation, social exclusion and various psychological effects ranging from low self-esteem to depression and suicidal ideation. Multiple comorbidities, cardiovascular, ocular, metabolic syndrome, obesity, insulin resistance, fatty liver disease, numerous autoimmune diseases, thyroid dysfunction and many others are associated with psoriasis.

The pathogenesis of psoriasis is extremely complex and not perfectly understood. Nevertheless, activation of the IL-23/IL-17 axis plays a pivotal role. IL-23 receptors rely on JAK2/TYK2 heterodimer-mediated signalling, implicating their role in the pathogenesis of the disease.^23^

### Tofacitinib

Tofacitinib, already approved for psoriatic arthritis by the U.S. Food and Drug Administration (FDA) since December 2017, is the best studied JAK inhibitor in psoriasis. 14 trials have been completed (two phase-1, five phase-2 and seven phase-3). The first randomized controlled trial (RCT) of topical tofacitinib included 71 mild-to-moderate psoriasis patients and studied two kinds of tofacitinib ointment (2% and 2% with penetration enhancer) for four weeks to a fixed area containing a psoriatic plaque. Ointment 1 (2% + penetration enhancer) decreased Target Plaque Severity Score by 54.4% in comparison with 41.5% of the control ointment.^24^

Another phase 2b study evaluated 1% and 2% topical tofacitinib ointment, once or twice daily, in 435 patients with mild-to-moderate plaque psoriasis. 17.9% of the patients receiving 2% tofacitinib at week 8, and 23% at week 12, versus 8.3% at week 8 and 8.8% at week 12 for vehicle, achieved a 75% reduction from baseline Psoriasis Area and Severity Index (PASI) score. Itch and Dermatology Life Quality Index (DLQI) also improved with tofacitinib compared to vehicle.

In respect to oral tofacitinib, the first phase 1, randomized, dose-escalation trial, was conducted to 59 patients for 14 days. Dosages were 5, 10, 20, 30 and 50 mg twice-daily or 60 mg once daily. All groups, except the 5mg, had a dose-dependent improvement of psoriasis compared to placebo.^25^

Other important studies include a 12-week comparison between tofacitinib 5 and 10 mg b.i.d, etanercept 50mg twice weekly and placebo in 1106 patients. 75% reduction in PASI score rates were 39.5% and 63.6% for the 5 and 10 mg tofacitinib groups and 58.8% for the etanercept group.^26^ Similar results were shown in a long-term extension study in 2867 patients. Through month 54, among those patients remaining in the study at each time point, PASI 75 response was achieved by 56–74% and PASI 90 response by 35–47% of the patients. During this study, 29 patients died, with nine deaths considered as potentially related to the drug. General and serious AEs were reported in 82.5% and 13.7% patients, respectively.^27^

There were also two similarly designed, phase 3 RCTs (OPT Pivotal 1 and 2) in which 745 patients received tofacitinib 5mg, 741 received 10mg, and 373 received placebo b.i.d. PASI 75 achievement rates were: OPT Pivotal 1 – 39.9%, 59.2% and 6.2% for tofacitinib 5 and 10 mg twice daily and placebo; OPT Pivotal 2 – 46%, 59.6% and 11.4% respectively.^28^

In general, AEs were similar across studies with infections being the most common. Rates were also similar, with around 80% of patients developing an AE and 10% discontinuing treatment because of them. The short and long-term safety profile of tofacitinib was stable over time and consistent between phase 3 studies. Regarding the different dosage regimens, tofacitinib 10 mg did not appear to be associated with higher rates of AEs or discontinuations due to AEs than the 5mg dosage.

### Ruxolitinib

Three phase 2 studies have been completed for topical ruxolitinib in psoriasis. In the first one, the drug showed better results than placebo and comparable to topical calcipotriene and betamethasone.^29^ Owing to the small population of 29 patients, statistically significant conclusions could not be reached. The same group of investigators conducted a bigger study with 200 patients where ruxolitinib 1% induced 40% mean PASI improvement compared to 1% with placebo.^30^

The most frequent AE of topical ruxolitinib was irritation in the site of application. No phase 3 trials of the drug are currently active.

### Other JAK Inhibitors

Baricitinib showed encouraging results in the only phase 2 study of the drug in moderate-to-severe psoriasis. 271 patients were randomized in five groups (baricitinib 2, 4, 8, 10mg and placebo). 43% of the patients in the 8mg group and 54% in the 10mg group achieved PASI 75 compared to 17% in the placebo group.^31^ Peficitinib also showed good dose-dependent results without serious AEs.^32^ Finally, no results have been published from the only trial of itacitinib in psoriasis.

## VITILIGO

Vitiligo is the most common acquired chronic pigmentation disorder. It is characterized by progressive epidermal melanocyte destruction, presenting as white patches, with a prevalence of approximately 1%.^33^

Vitiligo has devastating psychological effects to the patients, especially to women and people with dark skin in whom it is more easily noticeable.^34^ Common comorbidities include thyroid dysfunction, diabetes mellitus, alopecia areata and more. Contrary to psoriasis and AD, existing therapies for vitiligo have limited efficacy and the course of the disease is highly unpredictable, although spontaneous repigmentation rarely occurs.

Melanocytes are destroyed by CD8 T cells. Those cells produce Interferon-γ (IFN-γ) which plays a crucial role in the disease pathogenesis, accumulating more T cells in vitiligo lesions.^23^ Since IFN-γ signalling utilizes the JAK-STAT pathway, JAK inhibitors may be a step in the direction of much needed, new, safe and effective vitiligo treatments.

Currently, only ruxolitinib has been trialled for vitiligo. 11 patients applied 1.5% cream twice daily for 20 weeks. In facial lesions, Vitiligo Area Scoring Index was improved by 76%, but in other areas, the response was minimal. AEs were minor (erythema, hyperpigmentation, transient acne).^35^ It is unknown if better response in facial lesions was due to thinner epidermis of the face, or if light is important for topical JAK inhibitors. A subsequent report of two cases suggested the later.^36^

Three large trials, including the phase 3 TRuE-V1 and TRuE-V2 with 300 participants each, are expected to end in 2021.

**Table 2.** JAK inhibitors in dermatologic conditions, route of administration, selectivity and phase of most advanced trial.

**STUDY DRUG**	**RoA**	**SELECTIVITY**	**INDICATIONS**	**STUDY PHASE & STATUS**
Tofacitinib	O	JAK-3,1,>>2	PsO	Phase 3 completed
			AA	Phase 4 ongoing

Tofacitinib	T		AD	Phase 2 completed
			PsO	Phase 3 completed

Ruxolitinib	O	JAK-1,2	AA	Phase 2 completed

Ruxolitinib	T		PsO	Phase 2 completed
			vitiligo	Phase 3 ongoing
			AD	Phase 3 ongoing

Baricitinib	O	JAK-1,2	PsO	Phase 2 completed
			AA	Phase 3 ongoing
			AD	Phase 3 completed
			ACD	Phase 1 ongoing

Upadacitinib	O	JAK-1	AD	Phase 3 ongoing

Delgocitinib	T	pan-JAK	AD	Phase 2 ongoing
			DLE	Phase 2 ongoing
			CHE	Phase 2 ongoing

Abrocitinib	O	JAK-1	AD	Phase 3 ongoing

Gusacitinib	O	pan-JAK, SYK	AD	Phase 2 ongoing

Cerdulatinib	T	pan-JAK, SYK	Vitiligo	Phase 2 ongoing

Peficitinib	O	pan-JAK	PsO	Phase 2 completed

Itacitinib	O	JAK-1,2	PsO	Phase 2 completed
			Melanoma	Phase 1 ongoing
			Chronic itch	Phase 2 withdrawn

Filgotinib	O	JAK-1	CLE	Phase 2 completed

Lestaurtinib	O	JAK-2	PsO	Phase 2 completed

PF-06651600	O	JAK-3	AA	Phase 3 ongoing
			Vitiligo	Phase 2 ongoing

BMS-986165	O	TYK2	PsO	Phase 3 ongoing

RoA: route of administration, O: oral, T: topical, PsO: psoriasis, AA: alopecia areata, AD: atopic dermatitis, ACD: allergic contact dermatitis, DLE: discoid lupus erythematosus, CLE: cutaneous lupus erythematosus, CHE: chronic hand eczema.

## ALOPECIA AREATA

Alopecia Areata (AA) is another immune-mediated dermatological disease. It affects nearly 2% of the population and it is the second most common non-scarring alopecia after male and female pattern alopecia.^37^ It usually manifests as solitary, round, small patches of hair loss. At the other end of the disease spectrum, all hair in the scalp (alopecia totalis – AT) or whole body (alopecia universalis – AU) may be affected. Usually, treatment-resistance depends on the extent of the disease.

IFN-γ and Interleukin-15 (IL-15) signalling play a crucial role in AA development and maintenance. The first signals through JAK1/2 and the second mostly through JAK1/3.^38^ Thus, blocking the activation of these receptors may be a treatment option for AA.

### Tofacitinib

The largest published report is a retrospective study of 90 patients^39^ with severe disease (AA with more than 40% of scalp hair lost, AT or AU). Analysis of response rates yielded a critical period of over than 10 years duration of AT or AU to be the point at which patients were much less likely to respond to treatment. Patients not belonging in this group (n=65) were considered potential responders (AA independent of disease duration or AT/AU with duration of less than 10 years). 28 received tofacitinib 5mg b.i.d. and 37 adjuvant therapy (5mg b.i.d. with prednisolone, >5mg b.i.d, alone or with prednisolone) for 4 to 18 months. The clinical response rate was 77%. Response was better in patients with AA than those with AT/AU. No serious AEs were reported, with upper respiratory infection (28.9%) and headache (14.4%) being the most common.

In the group with more than 10 years duration of AT/AU, 68% showed no response to tofacitinib.

In an additional study with 66 patients,^40^ 32% achieved 50% or more improvement in the severity of alopecia with 5mg tofacitinib b.i.d. Again, AEs were minimal, mainly infections, and notably drug cessation resulted in disease relapse in two months.

In a Korean retrospective study,^41^ 32 patients with AA, AT or AU, refractory to previous treatments, again received 10mg tofacitinib daily. 24 patients had >5% hair regrowth (6 with 5–50%, 9 with 50%–90%, 9 with >90%) and 8 had no response. Finally, Serdaroğlu et al.^42^ reported a series of 63 patients who received tofacitinib 5mg b.i.d. for 12 months. 52 patients had >50% change in Severity of Alopecia Tool (SALT) score, with 25 of these having a complete response (>90% change in SALT score)

There are also many case reports and small case series showing the efficacy of tofacitinib in AA. Unfortunately, in most cases, discontinuation of the drug leads to disease relapse. Tofacitinib was also tested as a topical formulation in some case reports and small trials with poor results. In the largest study, with 16 patients, topical clobetasol dipropionate showed better results than tofacitinib.^43^

### Ruxolitinib

Ruxolitinib (20 mg b.i.d) was given as part of an open-label study^44^ to 12 patients. A significant hair regrowth, 92% from baseline on average, was observed in 9 patients after 3 to 6 months. Liu et al.^45^ administered ruxolitinib 10–25mg twice daily on 8 patients. 5 of 8 achieved complete or near-complete hair regrowth with a mean improvement in SALT score of 98%.

During the recent 28^th^ European Academy of Dermatology and Venereology (EADV) Annual Congress in Madrid, Spain, results from a phase 2 trial of CTP-543 (a deuterium-modified form of ruxolitinib) were presented.^46^ 58% of patients in the 12 mg twice-daily arm achieved a ≥ 50% relative reduction in their SALT score from baseline compared to 9% for placebo, with statistically significant separation from placebo occurring at Week 12. In the 8 mg twice-daily arm, 47% of patients achieved the primary endpoint compared to placebo. For the 4 mg cohort, there was not a statistically significant difference from placebo.

On the other hand, topical ruxolitinib seems to be less efficient than systemic. In the already mentioned study by Bokhari et al.,^43^ where topical clobetasol dipropionate was compared with topical JAK inhibitors, 5 out of 16 patients had partial regrowth in the areas treated with ruxolitinib 1% ointment.

### Baricitinib

The efficacy of oral baricitinib in AA has been demonstrated only in two case reports,^47,48^ but two large phase 3 trials (BRAVE-AA1 and BRAVE-AA2 with 725 and 476 patients) are currently ongoing.

### Other JAK Inhibitors

Several studies, small case series and case reports suggest that JAK inhibitors may be a promising treatment for numerous other dermatological disorders as dermatomyositis, cutaneous lupus erythematosus, chronic actinic dermatitis, erythema multiforme, hypereosinophilic syndrome, cutaneous graft-versus-host disease, allergic contact dermatitis, lichen planus, B cell mediated disorders, pyoderma gangrenosum and others.^49^

JAK inhibitors as a possible treatment for dermatologic diseases is a matter under intense research, but still a novelty; none have yet been approved for human use. Data from trials and experience from other medical specialties are encouraging, although some concerns arise. Cost of treatment is high, response is usually lost after treatment is stopped, optimal dosage, target and treatment duration are not precisely known and, most importantly, long-term safety results in dermatologic conditions are non-existent. Since JAK inhibitors are relatively new, real-life long-term safety data have just begun to emerge, although they concern non-dermatologic conditions, in which their usage has begun earlier.

Future research of JAK inhibitors in dermatology, beyond the above concerns, has clear directions in which it needs to move. As our understanding of the kinase family broadens, targeting will thankfully become more specific, increasing the precision of therapy, and thus lowering off-target activity and AEs. Finally, the easy accessibility of skin should be taken advantage of. As most topical treatments, future efficient topical JAK inhibitors will provide their benefits to the patients with lower cost and increased safety.

Concerning the already established biologics agents and the reasonable question as to how they compare to JAK inhibitors in the treatment of dermatologic disorders, we believe it is too early for a sensible answer. From the very limited existing data concerning non-dermatologic conditions, mainly RA, it seems that JAK inhibitors have comparable efficacy to biologics. Small (or, less likely, big) differences in efficacy will appear in future studies and answer this question. Until then, we can point out that, compared to biologics, JAK inhibitors are not proteins, so they do not trigger antidrug immune response, have wider blockade spectrum, are administered topically or orally, and are small molecules that are more easily made, hence, are more economical. Therefore, in our opinion, at this time, the important point is that we will soon have a different, new, promising weapon in our armamentarium against chronic, life-altering diseases.

## ABBREVIATIONS

AA: Alopecia areata
AD: Atopic dermatitis
AT: Alopecia totalis
AU: Alopecia universalis
BSA: Body surface area
DLQI: Dermatology Life Quality Index
EADV: European Academy of Dermatology and Venereology
EASI: Eczema Area and Severity Index
GM-CSF: Granulocyte-macrophage colony-stimulating factor
IFN: Interferon
IL: Interleukin
JAK: Janus kinase
PASI: Psoriasis Area and Severity Index
PGA: Physician Global Assessment
PsA: Psoriatic arthritis
RA: Rheumatoid arthritis
RCT: Randomized controlled trial
SALT: Severity of Alopecia Tool
STAT: Signal transducer and activator of transcription
SYK: Spleen tyrosine kinase
TYK2: Tyrosine kinase 2
